# Identification of biallelic variations of *CEP70* in patients with male infertility

**DOI:** 10.3389/fendo.2023.1133222

**Published:** 2023-03-09

**Authors:** Tiechao Ruan, Yihong Yang, Chuan Jiang, Gan Shen, Dingming Li, Ying Shen

**Affiliations:** ^1^ Department of Pediatrics, West China Second University Hospital, Sichuan University, Chengdu, China; ^2^ Key Laboratory of Obstetrics and Gynecologic and Pediatric Diseases and Birth Defects of the Ministry of Education, Sichuan University, Chengdu, China; ^3^ Reproduction Medical Center of West China Second University Hospital, Key Laboratory of Obstetric, Gynecologic and Pediatric Diseases and Birth Defects of Ministry of Education, Sichuan University, Chengdu, China; ^4^ Human Sperm Bank, Key Laboratory of Birth Defects and Related Diseases of Women and Children (Sichuan University), Ministry of Education, West China Second University Hospital, Sichuan University, Chengdu, China

**Keywords:** male infertility, oligoasthenoteratozoospermia, recessive inheritance, *CEP70*, WES

## Abstract

**Introduction:**

Male infertility is a severe health issue caused by complex and multifactorial pathological conditions. Genetic factors are a major cause of male infertility. CEP70, a centrosomal protein, has been reported to play an important role in male reproduction in mice. However, the role of CEP70 in human male infertility is limited.

**Methods:**

Whole exome sequencing and Sanger sequencing were used to identify the genetic cause of the infertile patients. Papanicolaou staining, scanning electron microscopy and transmission electron microscopy were further conducted to explore morphological and ultrastructural defects in spermatozoa from the patient. Immunofluorescence staining was used to detect the pathogenicity of the identified variants and the particular expression of CEP70 in testis.

**Results:**

In this study, we identified biallelic mutations of *CEP70* in two unrelated infertile male individuals with oligoasthenoteratozoospermia that followed a recessive inheritance pattern. Papanicolaou staining, scanning electron microscopy and transmission electron microscopy showed that morphological and ultrastructural defects in the acrosome and flagellum of sperm from the patient in a pattern strikingly similar to that in *Cep70^−/−^* male mice. The results of immunofluorescence staining suggested that CEP70 was normally expressed in the acrosome and flagellum of control sperm but was hardly detected in the sperm of patient carrying *CEP70* variation. We also explored the particular expression pattern of CEP70 during spermatogenesis in humans and mice.

**Conclusions:**

Biallelic mutations of *CEP70* might be a novel genetic cause of human male infertility, which could potentially serve as a basis for genetic counseling and diagnosis of male infertility.

## Introduction

Infertility is a major health problem worldwide. More than 186 million people suffer from infertility, accounting for 8-12% of couples of reproductive age (1, 2). Among them, male infertility causes more than half of the childless cases worldwide (3). Male infertility is a complex multifactorial pathological state with a highly heterogeneous phenotype, including azoospermia or oligozoospermia, asthenozoospermia and teratozoospermia (4). The causes of male infertility can be related to abnormal hypothalamic-pituitary-axis function, disrupted spermatogenesis in quantity and quality, and obstruction or dysfunction of reproductive ducts (4). Importantly, genetic factors are suggested to be the major causes of male infertility, accounting for 15% of all cases (5). Identifying the genetic causes of male infertility has obvious clinical significance, as it can guide individualized treatment that may have an impact on the reproductive health of patients and their children (6). In recent decades, modern genomics tools, especially exome sequencing, have led to further developments in the study of genetic factors in male infertility (7). However, the genetic etiology remains unknown in approximately 50% of cases (5). Therefore, it is important to explore more genes associated with human infertility in future studies.

Centrosomal proteins are the active components of the centrosome and are essential to the centrosome function (8). The centrosome mediates mitosis and meiosis in the early stage of spermatogenesis, connects the sperm head and tail, forms the sperm tail, controls the movement of the sperm tail, and organizes the cytoskeleton of the zygote (9). Due to the vital role of the centrosome in spermatogenesis, studies have revealed that centrosome abnormalities lead to male infertility, such as oligozoospermia, asthenozoospermia and teratozoospermia (9, 10). Centrosomal protein 70 (CEP70) belongs to the centrosomal protein family and is highly expressed in human sperm according to single-cell transcriptome data from germ cells (11). CEP70 was first found in proteomics studies of the centrosome and was suggested to express in the sperm tail (12, 13). However, only one study has suggested that loss of CEP70 function is involved in male infertility (14). In that study, the authors found that CEP70 deficiency leads to male infertility in mice, which is associated with abnormalities in sperm flagellum, head and acrosome, and heterozygous mutations of *CEP70* in four azoospermia patients were detected (14). Therefore, the role of *CEP70* in human fertility needs to be further explored.

In the present study, we identified two novel biallelic variants in *CEP70* that are responsible for human oligoasthenoteratozoospermia. Specifically, the patient carrying the *CEP70* mutation exhibited various abnormalities in sperm flagella as well as a lack of acrosomes, which was consistent with *Cep70*-/- male mice. Our findings revealed an undiscovered recessive inheritance pattern of *CEP70* mutations in human male infertility, which may provide new genetic evidence for the diagnosis and treatment of male infertility cases.

## Materials and methods

### Study participants

A 28-year-old Han Chinese male (Patient 1) and a 32-year-old Han Chinese male (Patient 2) from two different nonconsanguineous families with primary infertility were recruited for this study. The probands’ parents and controls with normal fertility were also included in the study. The inclusion criteria for controls with normal fertility were as follows: they must have at least one offspring; the semen quality of normal controls must reach the reference values of human semen characteristics provided by the World Health Organization (WHO), including total sperm count ≥ 39 million per ejaculation, sperm concentration ≥ 15 million/ml, progressive sperm motility ≥ 32% and morphologically normal sperm ≥ 4% (1). According to human semen characteristics, male infertility is divided into azoospermia or oligozoospermia, asthenozoospermia and teratozoospermia. Oligoasthenoteratozoospermia was defined as total number of spermatozoa, and percentages of both progressively motile and morphologically normal spermatozoa, less than the reference limits provided by WHO. This study was approved by the Ethics Committee of the Second West China Hospital of Sichuan University (reference number: 202053). All participants in this study signed an informed consent form.

### Genetic studies

Genomic DNA was isolated from peripheral blood samples of the probands for whole-exome sequencing (WES) using the FitAmp Plasma/Serum DNA Isolation Kit (Axygen Scientific, Union City, San Francisco, CA, USA). Then, exome capture was conducted with an Agilent SureSelect Human All Exon V6 Kit and Illumina HiSeq 2500 platform. ANNOVAR was used for functional annotation. The data filtering of gene variations was screened by a variety of databases, including the 1000 Genomes Project, ExAC, and HGMD databases. Furthermore, the pathogenicity of nonsynonymous variations was analyzed by SIFT, Mutation Taster, PolyPhen-2, and CADD software. Finally, the variations were selected according to the literature and public databases, including the National Center for Biotechnology Information (NCBI, https://www.ncbi.nlm.nih.gov), Human Protein Atlas (http://www.proteinatlas.org), and Mouse Genome Informatics (MGI, http://www.informatics.jax.org).

The candidate pathogenic variants were further validated in the patients and their healthy parents by Sanger sequencing. The PCR primers were as follows: Proband 1-F 5’-ATCAGATGCAAGAACCCAAAGTT- 3’, Proband 1-R5’-CTGCACATAAGACTGGTCACAA- 3’; Proband 2-F 5’-TTTTCCCAGCATTTCAGGCA- 3’, Proband 2-R 5’- GCATGGACAGAATGATGCCA- 3’.

### Papanicolaou staining

Semen samples fixed in 4% paraformaldehyde were coated on slides and air-dried. The slides were rehydrated with 80%, 70%, 50% ethanol and distilled water and stained with Lea’s hematoxylin. Then, the slides were rinsed with distilled water and stained with G-6 orange stain and EA-50. Finally, the slides were dehydrated with ethanol and mounted.

### Electron microscopy

The spermatozoa from the patient and control were imaged with scanning electron microscopy (SEM) and transmission electron microscopy (TEM). For SEM, the spermatozoa were prefixed in 2.5% glutaraldehyde at 4°C overnight. After washing in 1x phosphate-buffered saline (PBS) three times, the spermatozoa were dehydrated in progressive concentrations of ethanol (35, 50, 75, 90, 95, and 100%, 10 min each) and dried by a CO2 critical-point dryer (Eiko HCP-2, Hitachi). Finally, the spermatozoa were imaged using SEM (Hitachi S3400) at an accelerating voltage of 15 kV.

For TEM, the spermatozoa were fixed in electron microscopy fixative and postfixed with 1% OsO4. After dehydration with gradient acetone solutions, the spermatozoa were embedded in Epon 812. Ultrathin sections were obtained by an Em UC6 Ultramicrotome (Leica) and double stained with lead citrate and uranyl acetate. The sections were imaged using TEM (TECNAI G2 F20, Philips) at 120 kV.

### Immunofluorescence staining

For spermatozoa staining, the sperm of humans and mice were fixed with 4% paraformaldehyde solution and coated on slides. The spermatozoa were permeabilized with 0.3% Triton X-100 and blocked with 5% bovine serum albumin (BSA) solution. In regard to peanut agglutinin (PNA) immunofluorescence staining, 30% donkey serum was used for blocking instead of 5% BSA. Then, the spermatozoa were incubated with primary antibodies, including anti-CEP70 (1:50, 16280-1-AP, Proteintech) and anti-alpha-tubulin (1:1000, ab7291, Abcam), overnight at 4°C. After washing in 1x PBS (3 × 10 min), the spermatozoa were incubated with Alexa Fluor 488 (1:1000, 2309139, Thermo Fisher Scientific), Alexa Fluor 594 (1:1000, 2160431, Thermo Fisher Scientific), 4,6-diamidino-2-phenylindole (DAPI, 28718-90-3, Sigma−Aldrich) and PNA (1:50, 2328948, Thermo Fisher Scientific) for 2 h at room temperature. The spermatozoa were imaged with a laser scanning confocal microscope (Olympus, FV3000).

For testicular tissue staining, the testicular tissue of the adult mice was fixed with 4% paraformaldehyde solution. Then, the tissue was embedded in paraffin and cut into sections. After deparaffinization and rehydration, the tissue sections were boiled with 20 mM sodium citrate for 15 min at 95°C. Next, the tissue sections were incubated with the primary antibody anti-CEP70 (1:50, 16280-1-AP, Proteintech) overnight at 4°C, followed by incubation with Alexa Fluor 594 and DAPI for 2 h at room temperature.

### RNA isolation and quantitative real-time PCR

Total RNA of mouse tissues was extracted using TRIzol reagent (Invitrogen) and was reverse transcribed into cDNA using the PrimeScript RT reagent Kit (Takara). qRT−PCR was conducted using iTaq Universal SYBR Green Supermix (Bio-Rad Laboratories) on an iCycler RT−PCR Detection System (Bio-Rad Laboratories). Each test for each sample was performed in triplicate. In addition, actin was used as an internal reference, and the qRT−PCR data were normalized using the 2−ΔΔCt method. The primer sequences for real-time PCR were as follows: *Actin*-F 5’-CCTAGGCACCAGGGTGTGAT- 3’, *Actin*-R 5’ -TCACGGTTGGCCTTAGGGTT- 3’; *Cep70*-F 5’ -GCCCCAAACGGCAATAAAGA- 3’, *Cep70*-R 5’ - CTCCGACTTTGACACCTTCCT- 3’.

### Statistical analysis

Statistical analysis was conducted using GraphPad Prism 9.0.0 (GraphPad Software Inc, USA) and SPSS 17.0 (IBM Statistics, USA). Statistical significance between two groups was determined using an unpaired two-tailed Student’s *t* test. The P value of less than 0.05 was considered statistically significant.

## Results

### Identification of biallelic *CEP70* variants in two infertile males with oligoasthenoteratozoospermia

**Figure 1:**
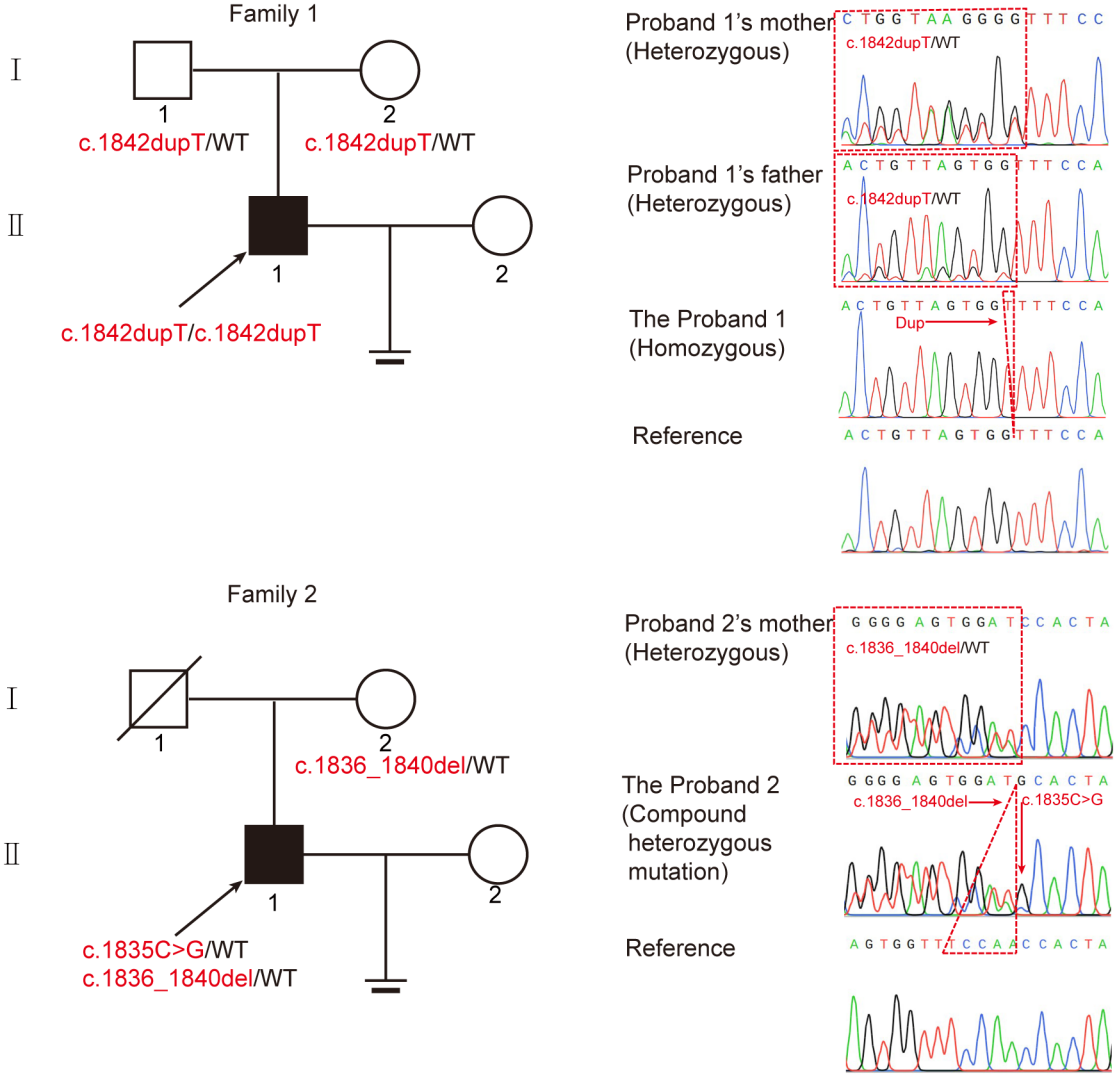
Identification of biallelic *CEP70* variants in two infertile males with oligoasthenoteratozoospermia from different families. The pedigree of two families with biallelic *CEP70* variants. The black arrow points to the probands. Sanger sequencing analysis from the two families. The red arrow points to the location of the mutation site. The red dotted triangle represents a duplicated or deleted base sequence.

Two infertile males were recruited for this study (**Figure 1**). The results of their semen analysis are shown in **Table 1**. The sperm count and motility were reduced significantly, and the malformed sperm morphology was considerably obvious. WES further revealed two biallelic mutations in *CEP70* in the two affected individuals (Supplementary Data 1 and 2). Noticeably, a homozygous frameshift variant of c.1842dupT (p. Pro615Thrfs*14) was identified in patient 1. This variant was absent or rare in most human populations according to the 1000 Genomes Project (0), ExAC Browser (0) and gnomAD databases (0.028%). For patient 2, compound heterozygous variants of c.1058C>G (p.Gly353Ala) and c.1059_1063del (p.Trp354Thrfs*14) were identified. These variants are both absent in the 1000 Genomes Project and ExAC Browser databases and are rare in the gnomAD database (0.025% and 0.025%, respectively).

**Table 1:** Results of the semen analysis and sperm morphology examination of the patients.

Parameter	Patient 1 (n=2)	Patient 2 (n=3)	Reference
Semen volume, ml	4.4 ± 0.57	4.3 ± 0.5	≥1.5
Sperm concentration, million/ml	7.4 ± 0.1	10.4 ± 6.7	≥15
Vitality, %	39.5 ± 29.0	49 ± 25	≥58
Motility, %	9 ± 1.4	3.7 ± 3.1	≥32
Abnormal head (%)	99.6 ± 0.6	99.1 ± 0.1	–
Abnormal head-tail conjunction (%)	9.4 ± 6.2	5.9 ± 5.7	–
Abnormal flagella (%)	82.2 ± 3.3	81.3 ± 2.8	–

Data are presented as mean ± SD.

To further clarify the contribution of these variants, we verified these variants in the two families by Sanger sequencing. For patient 1, his healthy parents each carried a heterozygous variant of c.1842dupT (**Figure 1**). For patient 2, his fertile mother carried a heterozygous variant of c.1059_1063del, and we could not obtain a DNA sample from his father who was dead. Our findings suggest that *CEP70* mutations might be related to male infertility in a recessive inheritance pattern.

### The spermatozoa phenotype involved in CEP70-mutated men

**Figure 2:**
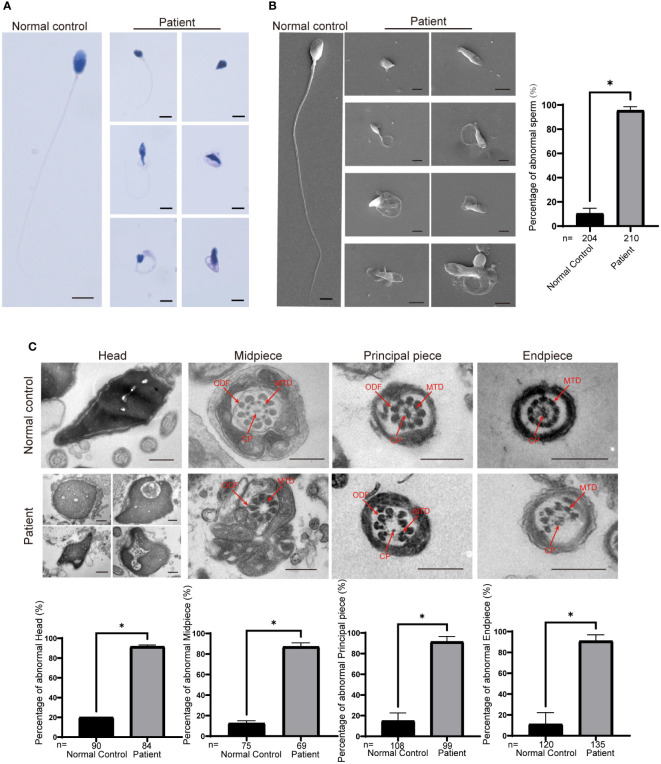
Morphological and ultrastructural defects in spermatozoa from the patient with *CEP70* mutation. **(A, B)** Morphological abnormalities in spermatozoa were observed from patient 1 by Papanicolaou staining **(A)** and SEM **(B)** (scale bars, 2.5 µm). ​Significant increases in the percentage of abnormal morphology in sperm from patient 1 (two-sided Student’s t test; *P < 0.05; error bars, s.e.m.). n, the number of sperm analyzed. **(C)** The deformed ultrastructure of spermatozoa was analyzed by TEM. The spermatozoa head of patient 1 was irregular and contained large vacuoles. The acrosome was deformed or even absent. The CP, MTD and ODFs were incomplete, disorganized and/or absent in the flagella of spermatozoa. CP, central-pair microtubules; MTD, peripheral microtubule doublets; ODF, outer dense fiber (scale bars, 500 nm). The percentage of ultrastructural defects in the head, midpiece, principal piece and endpiece in normal control and the patient sperm (two-sided Student’s t test; *P < 0.05; error bars, s.e.m.). n, the number of cross sections analyzed.

We next investigated the abnormal morphology of spermatozoa in patient 1. Regrettably, patient 2 refused to provide his semen for further research. Using the analysis of Papanicolaou staining and SEM, we observed that most of the spermatozoa had round, pyriform, tapered, or amorphous heads and a mosaic of flagellar morphological abnormalities, including absent, short, bent, coiled, and irregular tails (**Figures 2A, B**). In addition, TEM was used to analyze the ultrastructure of the sperm from patient 1 and the normal control. The sperm nucleus contained large vacuoles with membranous structures; the acrosome showed irregularly shaped deformations or was absent (**Figure 2C**). Remarkably, we found ultrastructure defects in the flagella of sperm from patient 1. Compared with the normal control, the cross section of flagella showed a severely fuzzy or incomplete ‘9+2’ microtubule structure, including characteristics of missed central-pair microtubules, partially absent peripheral microtubule doublets, and incomplete and disorganized outer dense fibers (**Figure 2C**).

**Figure 3:**
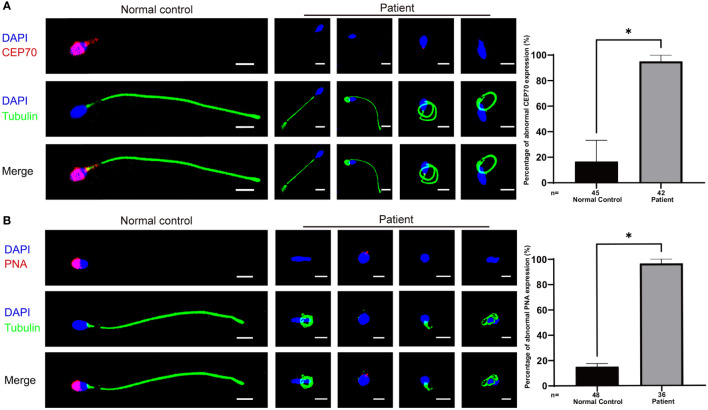
Immunofluorescence staining of CEP70 and PNA in patient sperm. **(A)** The immunofluorescence staining showed CEP70 protein localized in the acrosome and neck in the control sperm and was drastically decreased in spermatozoa from the patient (blue, DAPI; green, alpha-tubulin; red, CEP70; scale bars, 5 µm) Significantly abnormal expression of CEP70 in sperm from the *CEP70* mutation patient (two-sided Student’s t test; *P < 0.05; error bars, s.e.m.). **(B)** The PNA signal was defective in the sperm from patient 1 compared to the control sperm (blue, DAPI; green, alpha-tubulin; red, PNA; scale bars, 5 µm). Significantly abnormal expression of PNA in sperm from the *CEP70* mutation patient (two-sided Student’s t test; *P < 0.05; error bars, s.e.m.). n, the number of sperm analyzed.

We further assessed the effect of the c.1842dupT variant in *CEP70* on patient sperm by immunofluorescence staining. CEP70 mainly located in the sperm neck and acrosome in the normal control (**Figure 3A**). However, the CEP70 signal in patient sperm could not be detected (**Figure 3A**). The western blot using the sperm lysate of the patient also showed the CEP70 protein degradation (**Supplementary Figure 1**). In addition, PNA staining showed that patient 1 had severe defects in sperm acrosome formation compared to the normal control (**Figure 3B**). These findings suggest that CEP70 plays an important role in acrosomal and flagellar development during human spermatogenesis.

### The expression pattern of CEP70 in the testes of mice and humans

**Figure 4:**
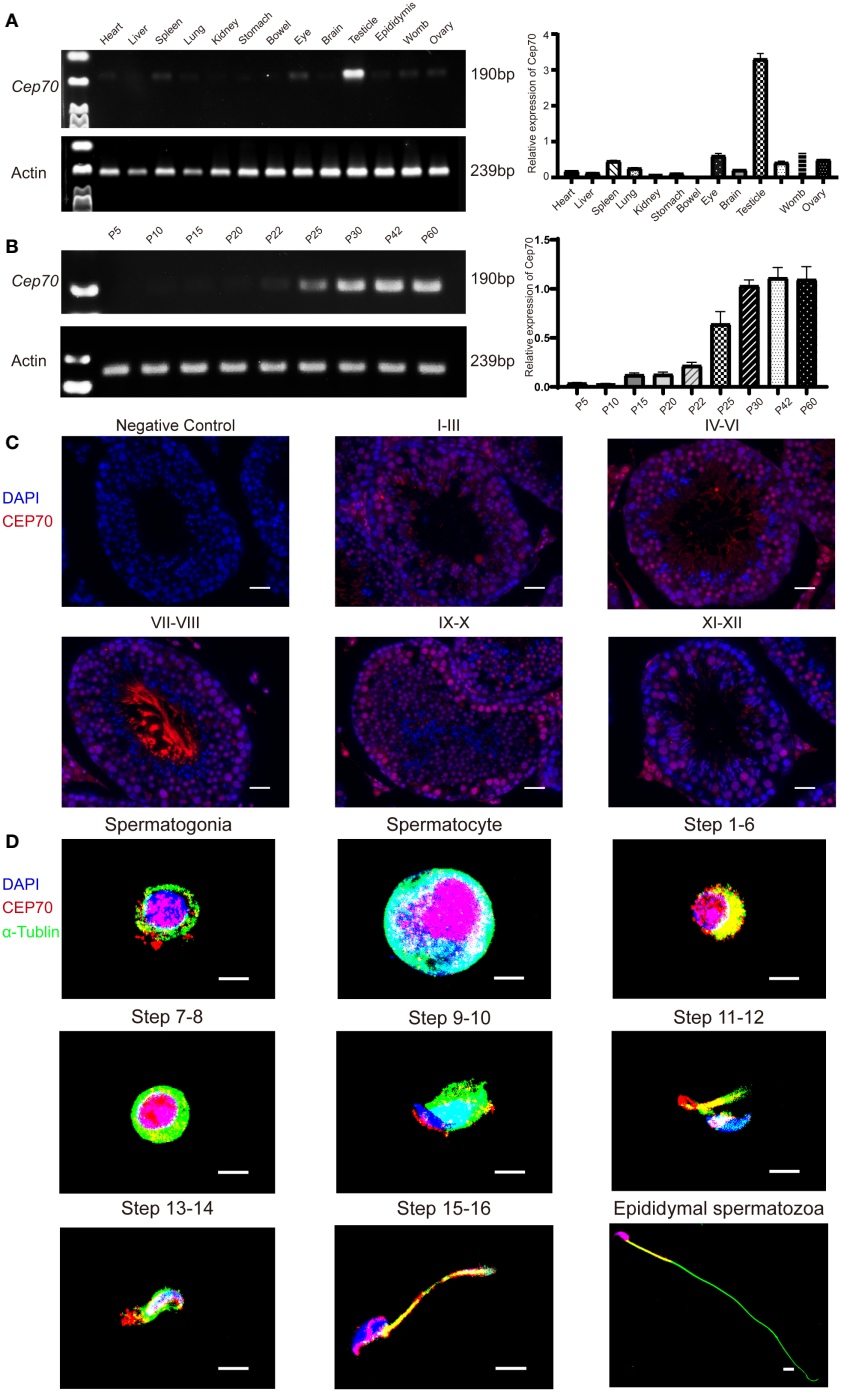
The expression pattern of CEP70 in the testes of mice. **(A)** *Cep70* dominantly expressed in the testes, as shown by qRT−PCR analysis. **(B)** qRT−PCR showed that *Cep70* began to express significantly on P25, reached its highest level on P30 and then showed a stable expression pattern (P= Postnatal day). **(C)** Immunofluorescence staining of CEP70 in different stages of mouse spermatogenesis (blue, DAPI; red, CEP70; scale bars, 5 µm). **(D)** Immunofluorescence staining of CEP70 in isolated germ cells (blue, DAPI; green, alpha-tubulin; red, CEP70; scale bars, 5 µm). Three independent experiments were performed.

We explored the expression levels of *Cep70* mRNA in various organs of adult mice using qRT−PCR to further identify the role of CEP70 in male reproduction. The qRT−PCR results showed that *Cep70* was mainly expressed in the mouse testis compared to other mouse organs, including the heart, liver, spleen, lung, kidney, stomach, bowel, eye, brain, epididymis, womb, and ovary (**Figure 4A**). In addition, we investigated the expression levels of *Cep70* in testicular tissues of mice at different postnatal days. The results showed that *Cep70* began to express obviously on postnatal Day 25, reached its highest level on postnatal Day 30, and then showed a stable expression pattern (**Figure 4B**). In addition, to better understand the roles of CEP70 during mouse spermatogenesis, we performed immunofluorescence staining of mouse testicular tissue. The results showed that CEP70 primarily located in spermatocytes, round spermatids, and elongated spermatids in testicular tissue (**Figure 4C**). Furthermore, we isolated various germ cells from mouse testes. The staining results showed that CEP70 mainly located in the nuclei and cytoplasm of spermatogonia, spermatocytes and round spermatids and was also expressed in the head and flagellum of late spermatids and epididymal spermatozoa (**Figure 4D**).

**Figure 5:**
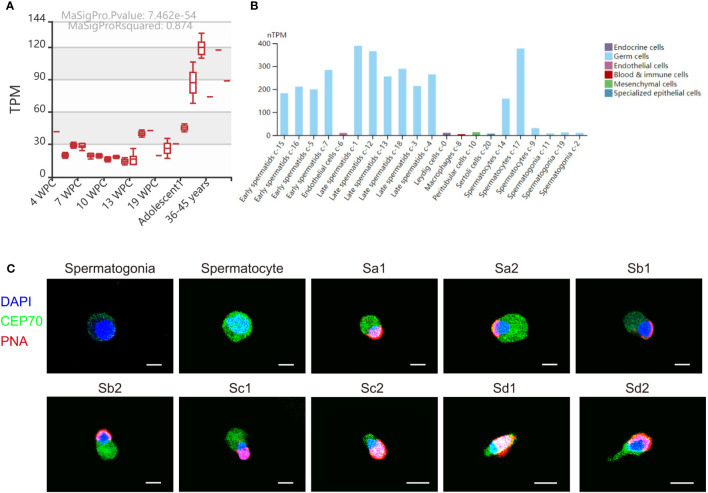
The expression pattern of CEP70 in human testes. **(A)** The RNA expression levels of *CEP70* at different developmental stages in human testes (TPM = transcripts per million; WPC = weeks postconception). **(B)** The expression levels of *CEP70* in different types of cells in human testes (https://www.proteinatlas.org/ENSG00000114107-CEP70/single+cell+type/testis). **(C)** Immunofluorescence staining of CEP70 in different stages of germ cells from human testis (blue, DAPI; green, CEP70; red, PNA; scale bars, 5 µm). CEP70 locates at the acrosome and flagella regions in sperm. Sa, Sa spermatid; Sb, Sb spermatid; Sc, Sc spermatid; Sd, Sd spermatid.

We also investigated the expression of *CEP70* in the process of spermatogenesis in humans. The results of the MeDas database (https://das.chenlulab.com) analysis showed that *CEP70* is mainly expressed in testicular tissue of adolescents and adults (**Figure 5A**). In addition, the results of single-cell sequencing in the Human Protein Atlas database showed that *CEP70* was mainly expressed in spermatocytes, early spermatids and late spermatids (**Figure 5B**). Moreover, we employed immunofluorescence staining to confirm the results provided in the above databases. The expression of CEP70 in spermatogonia was relatively low and was evident in spermatocytes. During spermiogenesis, CEP70 was present in the acrosome and flagella regions in spermatids, which was the same location as CEP70 in mice (**Figure 5C**). Overall, the current results suggest that CEP70 might play an important role in human male reproduction and influence acrosomal and flagellar formation.

## Discussion

Centrosomal proteins are closely associated with reproductive processes (15). However, evidence of a relationship between male infertility and centrosome protein functional defects is limited in humans. *CEP112* and *CEP135* deficiencies in humans lead to acephalic spermatozoa and multiple morphological abnormalities of the sperm flagella, respectively (16, 17). In addition, our recent findings suggest that CEP128 and CEP78 is involved in spermatogenesis in both humans and mice (10, 18). Intriguingly, in this study, biallelic mutations of *CEP70* in two infertile men followed a recessive inheritance pattern. Regrettably, patient 2 was unwilling to provide semen for further study, thus we only obtained the semen from patient 1 to explore the effect of *CEP70* mutations on sperm morphology. The infertile patient with *CEP70* mutations showed amorphous heads, abolished acrosomes, and anomalous flagella morphology in sperm. Our clinical report showed that CEP70 might play a vital role in human male reproduction.

Currently, there is limited information on the function of CEP70. CEP70 acts as a centrosomal protein that increases microtubule length and promotes microtubule stability through interaction with HDAC6 and regulation of microtubule protein acetylation (19, 20). Previous data showed that CEP70 is involved in cilia formation and determines the length of the axoneme in zebrafish embryos (21). A recent study suggested that *Cep70^−/−^* in mice caused male infertility, which resulted in abnormal acrosome structure and abnormal flagella (14). Furthermore, mice lacking CEP70 also exhibited disturbed spermiogenesis and increased germ-cell apoptosis, which led to a decrease in sperm count (14). Mechanistically, the previous study revealed that the expression of sperm flagellum development related proteins, including AKAP4, Tekt4, ODF1, CABYR, ROPN1 and TXNDC2 were decreased in *Cep70^−/−^* male mice by proteomics analysis (14). In addition, the reduced expression of the proteins involved in sperm acrosome formation was also detected in *Cep70^−/−^* mice, including AKAP3, ZP3R, SPACA1, ACRBP (14). Therefore, in our study, we also observed the abnormalities in sperm acrosomal and flagellar formation in the patient with biallelic *CEP70* mutations, which defects might be caused by the diminished expression of the acrosome and flagellum development related proteins suggested in *Cep70^−/−^* mice. However, Liu et al. reported a heterozygous mutation in *CEP70* in four azoospermia patients (14). Noticeably, *Cep70* ^+/−^ heterozygous mice have normal fertility to produce *Cep70^−/−^* mice. Moreover, our study showed that the heterozygous *CEP70* mutation was detected in the fertile father. In addition, the CEP70 protein is highly conserved in humans and mice. Therefore, it is plausible that the variants of CEP70 are recessively inherited to produce pathogenicity in male infertility.

In conclusion, the current study is the report of *CEP70* mutation-related male infertility in humans with a recessive inheritance pattern, similar to the mouse model. Future cases are needed to corroborate the proposed link of *CEP70* mutations with male infertility in order to use it as a target for genetic counseling and diagnosis of male infertility. We believe that this study will expand our understanding of the role of centrosomal proteins in human male infertility.

## Data availability statement

The datasets presented in the study are deposited in the MeDas online repository, https://das.chenlulab.com.

## Ethics statement

The studies involving human participants were reviewed and approved by the Ethics Committee of the Second West China Hospital of Sichuan University. The patients/participants provided their written informed consent to participate in this study. Written informed consent was obtained from the individual(s) for the publication of any potentially identifiable images or data included in this article.

## Author contributions

All authors contributed to the study conception and design. Sample collection whole-exome sequencing, and screening for the mutations were performed by YY and DL. TR, CJ, and GS performed the experiments and collected the data. The first draft of the manuscript was written by TR and YS revised the manuscript, and all authors commented on previous versions of the manuscript. All authors contributed to the article and approved the submitted version.

## Supplementary material

Supplementary Figure 1 The absence of CEP70 expression in patient’s sperm lyste. ​Western blotting detected loss of CEP70 protein in the sperm lysate from the patient compared to the normal control.
