# Supplementary figures and images for: Identification of biallelic variations of CEP70 in patients with male infertility
Source: Front Endocrinol (Lausanne). 2023 Mar 9;14:1133222. doi: 10.3389/fendo.2023.1133222 (PMC10035576; doi:10.3389/fendo.2023.1133222)

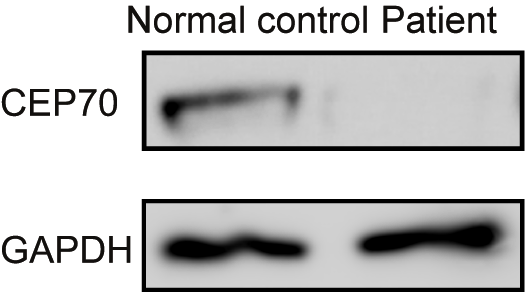

Supplement: Supplementary Figure 1 — The absence of CEP70 expression in patient’s sperm lyste. ​Western blotting detected loss of CEP70 protein in the sperm lysate from the patient compared to the normal control. [file Image_1.tif]
